# Thyroid Hypoplasia as a Cause of Congenital Hypothyroidism in Monozygotic Twins Concordant for Rubinstein-Taybi Syndrome.

**DOI:** 10.4274/jcrpe.v3i1.07

**Published:** 2011-02-23

**Authors:** Mustafa Ali Akın, Tamer Güneş, Leyla Akın, Dilek Çoban, Sena Kara Oncu, Aslıhan Kiraz, Selim Kurtoğlu

**Affiliations:** 1 Erciyes University, Faculty of Medicine, Department of Pediatrics, Division of Neonatology, Kayseri, Turkey; 2 Erciyes University, Faculty of Medicine, Department of Pediatrics, Division of Pediatric Endocrinology, Kayseri, Turkey; 3 Erciyes University, Faculty of Medicine, Department of Pediatrics, Kayseri, Turkey; 4 Erciyes University, Faculty of Medicine, Department of Medical Genetics, Kayseri, Turkey; 5 Erciyes University, Faculty of Medicine, Department of Pediatrics, Division of Pediatric Endocrinology and Neonatology, Kayseri, Turkey; +90 532 561 79 45 mustafaaliakin@hotmail.com Erciyes University, Faculty of Medicine, Department of Pediatrics, Division of Neonatology, Melikgazi, Kayseri, Turkey

**Keywords:** Rubinstein-Taybi syndrome, monozygotic twins, congenital hyperthyroidism, thyroid hypoplasia

## Abstract

Rubinstein-Taybi syndrome (RSTS), a genetic disorder characterized by growth retardation, mental deficiency, dysmorphic face, broad thumbs and large toes, generally affects monozygotic twins concordantly. Thyroid hypoplasia (TH) is a common cause of congenital hypothyroidism (CH) and often accompanies dysmorphic syndromes. A pair of female twins were admitted to our neonatology unit 16 hours after delivery. They were born at 35 weeks of gestation. Both twins had an unusual dysmorphic facial appearance with microcephaly, as well as broad short thumbs and large toes. Based on the presence of characteristic dysmorphic features, the twins were diagnosed as RSTS. Thyroid function tests in the first twin revealed the following results: free thyroxine (T4) 8.4 pg/mL, thyrotropin (TSH) 4.62 mIU/L, thyroglobulin (TG) 213.24 ng/mL and a normal level of urinary iodine excretion (UIE). Thyroid function test results in the second twin in the second week were: free T4 5.9 pg/mL, TSH 9.02 mIU/L, TG 204.87 ng/mL, and normal UIE levels. Thyroid volumes were 0.36 mL and 0.31 mL in the first and second twin, respectively. TH was confirmed by technetium 99 m pertechnetate thyroid scans in both infants. Thyroid function tests normalized with L-thyroxine replacement therapy (10 μg/kg/day) around the end of the 3^rd^ week of life. The infants were discharged planning their follow-up by both endocrinology and cardiology units. The rarity of cases of twins with RSTS (concordant) co-existing with CH led us to present this report.

**Conflict of interest:**None declared.

## INTRODUCTION

Rubinstein-Taybi syndrome (RSTS, OMIM 180849) is a rare condition, inherited in an autosomal dominant fashion and characterized by short stature, well-established craniofacial features, moderate to severe motor and intellectual disability, broad, often angulated thumbs, and enlarged first toes. The prevalence of RSTS is approximately one per 300 000 persons and may be as high as one per 1 000 live births (1,2,3,4,5,6). Most cases of RSTS are sporadic and families with more than one affected child are extremely rare. Apart from one of the monozygotic twins reported to date, this syndrome has been noted as concordant (3). The known genetic causes of RSTS are point mutations or microdeletions of the response element-binding protein-binding protein (CREBBP) gene encoding cAMP CREBBP, which is localized to 16p13.3 (50-60%), and of the EP300 gene encoding E1A binding protein p300 localized to 22q13.2 (5%) (1,2,3,4,5,6). 

Thyroid dysgenesis (TD) is a common cause of congenital hypothyroidism (CH), affecting about one in 5 000 live births. There are several reports of an increased frequency of birth defects associated with primary CH, but TD is generally considered as a nongenetic disease (7,8). Various endocrinological problems are expected in patients with mutations of CREBBP since they disrupt the hormonal mechanisms secondary to messenger cAMP (9). However, the only endocrinologic problem reported so far in patients with RSTS in neonatal period is CH without any abnormality in thyroid structure (10). Two cases of thyroid hypoplasia (TH) or TD as the cause of CH in monozygotic twin sisters concordant for RSTS will be presented in this report.    

## CASE REPORTS

**Figure 1:**
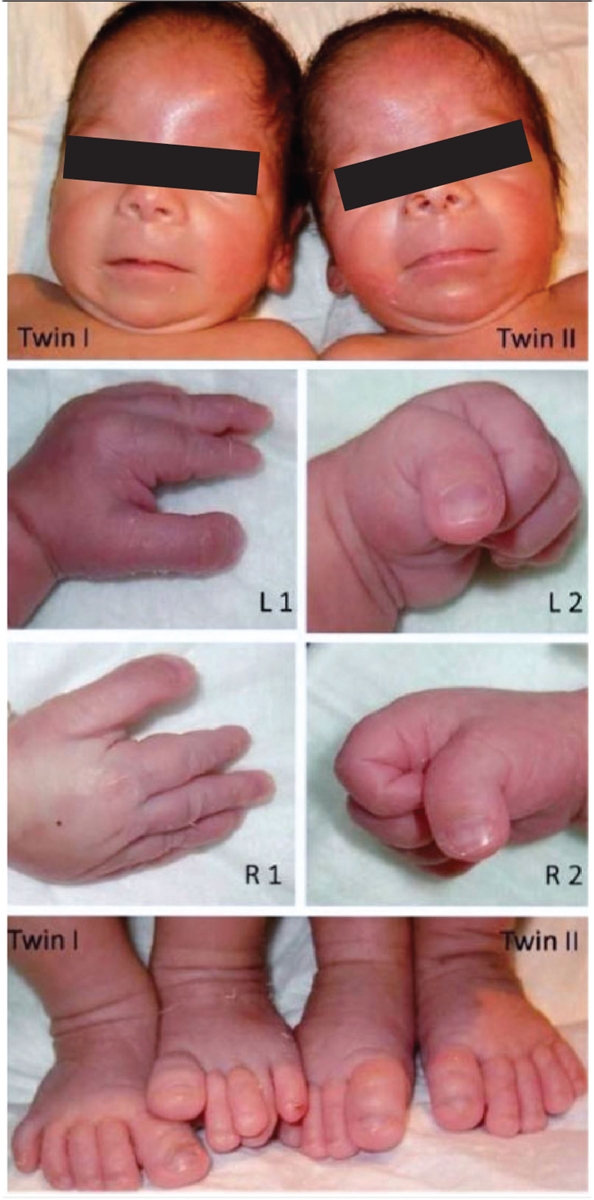
The appearance of the face, the broad, short thumbs and large toes are characteristic features of the Rubinstein-Taybi syndrome. The second twin had angulation of the distal interphalangeal joints of the thumbs. (L1-R1 and L2-R2 designate the left and right hands of first and second twins, respectively)

Twin sisters, aged 16 hours, were admitted to our Neonatology Unit due to respiratory distress. The twins were born by caesarean section at 35 weeks of gestation. They were the third and fourth children of healthy 32-year-old mother and 34-year-old father. There was no consanguinity between the parents. The mother had received adequate prenatal care, and monochorionic-diamniotic twin pregnancy was diagnosed in the second trimester of her pregnancy. At admission, both twins were cyanotic and tachypneic with intercostal retractions and grunting respirations. Clinical findings improved with administration of nasal continuous positive airway pressure. The first twin weighed 1900g (10-25^th^ centile), measured 43cm (10-25^th^ centile) in length and had a head circumference of 29cm (<10^th^ centile). Anthropometric measurements of the second twin yielded a birth weight of 1780g (10-25^th^ centile), length of 42cm (10-25^th^ centile), and head circumference of 28cm (<10^th^ centile).  The twins had an unusual dysmorphic facialappearance with microcephaly, prominent forehead, down-slanting palpebral  fissures, epicanthal folds, hyperteleorism, beaked nose, broad  nasal  bridge, hypoplastic  philtrum,  thin upper lip, swollen-thick lower lip and micrognathia, and a facial expression, which resembled an excruciating smile (Figure 1). Both sisters also had broad short thumbs and large toes. Additionally, the second twin had radial angulation of the distal interphalangeal joint of her thumbs. Based on the presence of characteristic dysmorphic facial features, broad thumbs and large toes, the twin sisters were diagnosed as RSTS.

Laboratory findings including hemoglobin, hematocrit, serum calcium, phosphorus, alkaline phosphatase, BUN, creatinine, electrolytes, blood sugar, ALT, AST, acid-base values, and urine analysis were normal. Both twins had the same blood group [A Rh (-)], consistent with monozygocyty. Thyroid function test results on day 7 in the first twin were: free thyroxine (T4) 8.4 pg/mL (normal: 11±3 pg/mL), thyrotropin (TSH) 4.62 mIU/L (normal: 3.8±2.3 mU/L), thyroglobulin (TG) 213.24 ng/mL (normal: up to 250 ng/mL) with normal level of urinary iodine excretion (UIE) 30 mg/dL (normal: higher than 10 mg/dL). At age two weeks, thyroid function test results in the second twin were: free T4 5.9 pg/mL (normal: 12±3 pg/mL), TSH 9.02 mIU/L (normal: 4.3±1.6 mU/L), TG 204.87 ng/mL (normal: up to 250 ng/mL), and UIE 40 mg/dL. Thyroid volumes were 0.36 mL and 0.31 mL (0.60±0.07 mL) (11), respectively (Table 1). TH was confirmed by technetium 99 m pertechnetate thyroid scans, and no ectopic tissue was observed in either twin (Figure 2).

Echocardiography was performed because of cardiac systolic murmur in both infants. Perimembranous ventricular septal defect (VSD) and patent foramen ovale were found in the first twin; perimembranous inlet VSD and a mild patent ductus arteriosus were detected in the second twin. 

Thyroid function tests normalized with L-thyroxine replacement therapy (10 μg/kg/day) around the end of the 3^rd^ week of life. Both infants were discharged and the follow-up was provided by both Pediatric Cardiology and Endocrinology units. The first one died at age 3 months as a result of aspiration pneumonia. The second twin completed the postnatal 5 months of life with poor neurological and somatic development.

**(Figure 2):**
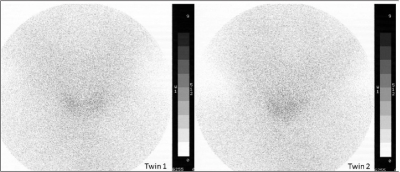
Thyroid hypoplasia without ectopic tissue was detected by technetium 99m pertechnetate thyroid scintigraphy in both twins

**(Table 1):**
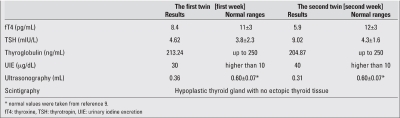
Results of thyroid function tests and imaging studies in the twins

## DISCUSSION

RSTS is a prototype of diseases with genetic heterogeneity. The characteristic craniofacial features of the syndrome are down-slanting palpebral fissures, columella extending below the nares, a highly arched palate, and a grimacing smile. Additional features of the disorder include eye abnormalities, heart and kidney defects, dental problems, and obesity. Prenatal growth is often normal; however, postnatal growth rate rapidly declines in the first few months of life. People with RSTS have an increased risk of developing benign and malignant tumors, including certain kinds of brain tumors and hematologic malignancies. The severity of these signs and symptoms vary among affected individuals (1,2,3,4,5,6,12,13,14). In the neonatal period, accompanying problems include respiratory and feeding difficulties, congenital heart defects, seizures and growth retardation. Individuals with RSTS are easily diagnosed early in life with their typical stigmata, as was the case in our patient (6,12,13,14). The severity of influence was concordant in our patients since they had similar phenotypic findings such as typical facial appearance, broad thumbs and toes, similar cardiologic defects, and a similar thyroid defect. 

CREBBP is a large nuclear protein, which plays anessential role in controlling cell growth and division, prompting cells to mature and assume specialized functions. It is also important for transcription regulation, chromatin remodeling, integration of several different signal transduction pathways including cAMP, nuclear hormone receptors, signal transducer and activator of transcription (STAT)  proteins, and for activating protein-1. In addition, it has been shown to be a critical co-activator for thyroid hormone receptors (7,8,9). On the other hand, the TSH and thyrotropin-releasing hormone (TRH) genes are positively regulated by cAMP that acts as a tonic stimulator of these hormone genes. Additionally, TSH stimulation increases mitotic activity in thyroid cells by raising the intracellular cAMP (9). Mutations of the CREBBP are expected to impair the tonic cAMP stimulation of TRH and TSH. For that reason, the faulty cAMP signaling decreases the basal TSH secretion and impairs the effects of TSH in thyroid cells (9). Probably, TH and hypothyroidism in patients with RSTS can be explained by the above-mentioned defective mechanism ongoing from intrauterine life.  Additionally, this mechanism is expected to cause resistance to thyroid hormones, and thus it may be associated with several features of RSTS, such as mental retardation, growth retardation and obesity (6,7,13).  

Endocrinologic disorders like premature thelarche (15) and obesity (13) during childhood or adolescence have been rarely reported in cases of RSTS, and only one case of CH has been described as yet (10). Developmental defects of the thyroid gland, such as absence, small size or unusual location, are collectively named TD. Although familial occurrences have been described, most cases of TD are sporadic and the etiology remains unclear (8,16,17). Recently, several gene defects of transcription factors {such as PAX8, NKX2-1 [encoding thyroid transcription factor (TTF)-1], FOXE1 (encoding TTF-2), NKX2-5 and thyroid-stimulating hormone receptor (TSHR)} have been determined among patients with permanent primary CH (8,16). Interestingly, TD is rarely observed in both of monozygotic twins. This finding may be associated with the influence of non-genetic factors (8,16,17). However, our monozygotic twins showed similar clinical findings of TD and RSTS. We speculate that this association may be more frequent in patients with RSTS who have CREBBP mutations due to the above-mentioned functions of this large protein.

In conclusion, we presented monozygotic twins with RSTS co-existing with TD and CH. To our knowledge, this is the first reported case of RSTS accompanied by CH in monozygotic twins. Since both disorders are very rare, this association may have been coincidental. However, we also considered the possibility that hypothyroidism resulting from TH might be a component of RSTS.  

## ACKNOWLEDGEMENT

Authors are thankful to Delbert A. Fisher from David Geffen School of Medicine at UCLA, Quest Diagnostics Inc., Quest Diagnostics Nichols Institute, San Juan Capistrano, CA-USA for reviewing the article. 
